# The diagnostic accuracy of mean platelet volume in differentiating immune thrombocytopenic purpura from hypo-productive thrombocytopenia: A systematic review and meta-analysis

**DOI:** 10.1371/journal.pone.0295011

**Published:** 2023-11-30

**Authors:** Muluken Walle, Mesay Arkew, Haftu Asmerom, Addisu Tesfaye, Fasil Getu

**Affiliations:** 1 Department of Medical Laboratory Sciences, College of Medicine and Health Sciences, Jigjiga University, Jigjiga, Ethiopia; 2 School of Medical Laboratory Sciences, College of Health and Medical Sciences, Haramaya University, Harar, Ethiopia; Saskatoon Cancer Centre and College of Medicine, University of Saskatchewan, CANADA

## Abstract

**Background:**

Thrombocytopenia is defined as a decreased number of platelets in the circulating blood as a result of hypo-proliferation in marrow or peripheral destruction of platelets. Several diagnostic methods have been proposed to discriminate the underline cause of thrombocytopenia. Recent studies showed that mean platelet volume (MPV) could be used for differential diagnosis of immune thrombocytopenic purpura (ITP). Thus, we aimed to investigate the diagnostic accuracy of MPV for differential diagnosis of ITP from hypo-productive thrombocytopenia.

**Methods:**

This study was conducted in accordance with the Preferred Reporting Items for Systematic Review and Meta-Analysis guidelines (PRISMA). The study protocol was registered on PROSPERO with the reference number CRD42023447789. Relevant published studies that were published up to April 10, 2023, in peer-reviewed journals were searched on electronic different databases. The methodological quality of the included studies was appraised using the quality assessment of diagnostic accuracy studies 2 (QADAS-2) tool. The pooled weight mean difference (WMD) of MPV between the ITP group and hypo-productive group was analyzed using a random-effects model meta-analysis. Relevant data were extracted using a Microsoft Excel spreadsheet and analyzed using STATA 11.0 and Meta-disc 1.4 software. Publication bias was evaluated using Deek’s funnel plot asymmetry test.

**Results:**

A total of 14 articles were included in this systematic review and meta-analysis. The comparison of MPV between groups revealed that the pooled mean value of MPV increased significantly in ITP patients compared to patients with hypo-productive thrombocytopenia (WMD = 2.03; 95% CI, 1.38–2.69). The pooled sensitivity and specificity of MPV in differentiating ITP from hypo-productive thrombocytopenia were 76.0% (95% CI: 71.0%, 80.0%) and 79.0% (95% CI: 75.0%, 83.0%), respectively. The summary positive likelihood ratio (PLR) and negative likelihood ratio (NLR)using the random effects model were 3.89 (95% CI: 2.49, 6.10) and 0.29 (95% CI: 0.18, 0.46), respectively.

**Conclusion:**

MPV can be used to discriminate ITP from hypo-productive thrombocytopenia. It can possess large advantages as it is noninvasive, simple, quick, inexpensive, easy to perform, reliable, and routinely generated by automated cell counters.

## 1. Introduction

Thrombocytopenia is defined as a decreased number of platelets less than 150 × 10^9^/L in the circulating blood [1]. Platelets are anucleated colorless blood cells that play an important role in primary hemostasis processes [2, 3]. Thrombocytopenia typically results in mucosal bleeding consequent to primary hemostasis defect [1]. It can be noticed in different clinical presentations including epistaxis, gingival bleeding, abnormal uterine bleeding, petechiae, and ecchymosis [4]. Thrombocytopenia can result from a variety of etiologies ranging from benign disorders to syndromes associated with significant morbidity [5].

The two main causes of thrombocytopenia excluding pseudo thrombocytopenia are hypo-proliferation in marrow and peripheral destruction of platelets. Hypo-proliferative bone marrow results in decreased platelet production called hypo-productive thrombocytopenia. It is associated with several bone marrow diseases [6] including, leukemia, aplastic anemia, myelodysplastic syndrome (MDS), and chemotherapy [2, 3]. Moreover, lymphoma, multiple myeloma, metastasis, carcinoma, and megaloblastic anemia can cause hypo-productive thrombocytopenia [7]. The second cause for the occurrence of thrombocytopenia is increased platelet destruction or peripheral consumption [6].

Immune thrombocytopenic purpura (ITP) is an autoimmune bleeding disorder that primarily manifests as an increased platelet destruction in the peripheral blood [8]. Production of specific IgG antiplatelet autoantibodies which are responsible for excessive platelet destruction and decreased platelet survival is thought to be the underlying cause of ITP [9]. Around 60%-80% of patients with ITP produce antibodies against platelet membrane glycoprotein (GP), mainly platelet membrane GPIba and GPIb/IX complex [10]. Genetic and acquired factors probably contribute to the production of antibodies against platelet surface receptors [11]. The commonly cited acquired events are viral infections and drugs. Antibodies against certain viral antigens may cross-react with normal platelet antigens (a form of molecular mimicry) [12, 13]. Drug-induced ITP may happen secondary to certain drug exposure which causes antibody-mediated accelerated platelet destruction [14]. Moreover, various factors including T-cell mediated or oxidative stress-dependent platelet destruction, and impaired megakaryopoiesis have been proposed to cause ITP [15].

Several diagnostic methods have been proposed to discriminate whether a low platelet count in a patient is caused by decreased production or increased destruction [16]. The gold standard method to discriminate causes of thrombocytopenia as hypo-productive or hyper-destructive is bone marrow examination. However, this approach is invasive, painful, time-consuming, uncomfortable, expensive, and unfriendly for the patients [1, 2]. Moreover, there have been prior reports on the role of platelet-associated immunoglobulin G (PAIgG) autoantibodies in platelet GP detection in the diagnosis and treatment of ITP [8]. However, antibody detection is an insufficient diagnostic test [17]. The main drawback to this approach lack of sensitivity, some patients may not have detectable antibodies at the time of diagnosis [8, 18]. Furthermore, anti-platelet antibody detection is not a specific test for ITP, the same antibodies may be found in diseases other than ITP [19]. As a result, antibody detection in plasma is not currently recommended [17]. Thus, a new non-invasive diagnostic approach for thrombocytopenia is needed [20].

Mean platelet volume (MPV) is among platelet indices that have been incorporated in the complete blood count (CBC) test with the technological advancement of automated hematologic analyzers [15]. It indicates the average size of platelets in the blood that provides vital information for megakaryopoietic activity [21]. A high MPV is associated with increased platelet production while a low MPV indicates decreased platelet production [22]. The biomarker role of MPV has been emphasized in different medical conditions including diabetes [23], metabolic syndrome [24], cancer [25], inflammatory bowel disease [26], preeclampsia [27], and ITP [16]. In ITP, an increased number of new platelets are formed in the bone marrow in response to excessive platelet destruction in the spleen. Then, the newly formed platelets which are younger and larger than the normal are released into the bloodstream, resulting in an increased MPV in ITP [28]. Therefore, MPV could help to distinguish ITP from hypo-productive thrombocytopenia [20]. Moreover, MPV possesses large advantages in differentiating the cause of thrombocytopenia because it is a noninvasive, simple, quick, cost-effective, easy-to-perform, and reliable marker [15]. Many studies supported the use of MPV for diagnosis of ITP [1, 15, 29], however, few studies recommended not to use MPV as screening test for ITP [30]. Thus, this study aimed to assess the summary diagnostic value of MPV in discriminating ITP from hypo-productive thrombocytopenia.

## 2. Methods

### 2.1. Study protocol

This systematic review and meta-analysis was conducted based on the updated 2020 preferred reporting item for systematic review and meta-analysis (PRISMA) guideline 2020 [31]. Moreover, the protocol of the study had been registered in the International Prospective Register of Systematic Reviews (PROSPERO) database (registration number: CRD42023447789). The study analyzed findings from published articles to evaluate the pooled weighted mean difference (WMD) of MPV between the ITP group and hypo-productive group globally and to investigate the diagnostic accuracy of MPV for differential diagnosis of thrombocytopenia.

### 2.2. Eligibility criteria

#### 2.2.1. Inclusion criteria

Studies that met the following criteria were considered eligible for inclusion in this study.

**Study design:** All published original studies with cross-sectional, case-control, and cohort study designs were included.

**Language and time restrictions:** We have included studies that were written in the English language and published online in peer-reviewed journals up to April 10, 2023.

**study participants:** We included studies that were carried out on patients with a primary clinical diagnosis of ITP and also having a hypo-thrombocytopenia group. Studies having Aplastic anemia, acute leukemia, chemotherapy, and MDS groups were considered hypo-productive groups.

**Study area:** Those studies that were conducted all over the world.

**Outcome measures:** Studies that have reported the value of MPV and expressed the results as mean and standard deviation (SD) or median and interquartile range (IQR) for both ITP from hypo-thrombocytopenia groups were included. Moreover, sufficient data on the diagnostic sensitivity and specificity of MPV in differentiating ITP from hypo-productive thrombocytopenia were important to include studies.

#### 2.2.2. Exclusion criteria

After a thorough screening of the abstracts and the full texts, the following studies were excluded.

- Studies with low methodological quality
- Studies that did not have both ITP and hypo-productive thrombocytopenia groups
- Studies that did not report the MPV value and/or the sensitivity and specificity of MPV
- Studies were case reports, reviews, poster presentations, and letters to the editor
- Studies which were published in other than English languages

### 2.3. Search strategy

A comprehensive search of previously published studies was performed on electronic databases including PubMed/MEDLINE, Science Direct, Cochrane Library, Scopus, Web of Science, and EMBASE. Moreover, reference lists probing published articles and searching through Google Scholar and Google were employed to identify additional relevant studies. This was performed by three reviewers (MW, HA, and AT). Medical Subject Headings (Mesh) terms and keywords in combination using Boolean operators like “OR” or “AND” were used to search relevant studies in the electronic databases. The searching terms used in searching were “Platelet Indices” or “Platelet parameters” or “Mean Platelet volume” or “MPV” AND “thrombocytopenia” or “Immune thrombocytopenia purpura” or “Idiopathic thrombocytopenia purpura” or “ITP”

### 2.4. Selection process

All identified articles through extensive searching were imported into EndNote X9 (Thomson Reuters, New York, USA) to organize and avoid duplicates. Then, two authors (MW and FG) independently and meticulously screened the titles and/or abstracts of each of the retrieved articles for eligibility. In case of possible arguments between the two review authors, mutual consensus was reached through discussions. Then, the two reviewers (MA and FG) independently appraise the full-text articles for inclusion. Again, any disagreements were solved through discussions and mutual consensus. If necessary, the third reviewer (HA) was involved to settle the final decision.

### 2.5. Data collection process

We extracted data from eligible studies using a Microsoft Excel spreadsheet data extraction form. The first author’s name, year of publication, study country, number of participants in each group, and the mean±SD values of MPV in each group were extracted. Results that were expressed as median and IQR were also extracted and changed to mean±SD using a method recommended by Hozo et al [32]. Moreover, the probability of sensitivity and specificity of MPV in predicting ITP for selected optimal cutoff values were extracted. Data such as true positives, false positives, false negatives, and true negatives were calculated. Finally, the extracted data were entered into a Microsoft Excel spreadsheet.

### 2.6. Outcomes of interest

The primary outcome of the study was determining the diagnostic performance of MPV in differentiating ITP from hypo-productive thrombocytopenia and also assessing the pooled mean difference value of MPV between ITP patients and patients with hypo-productive thrombocytopenia.

### 2.7. Quality assessment

The methodological quality of the included studies was appraised in detail using the quality assessment of diagnostic accuracy studies 2 (QUADAS-2) tool [33]. The tool consists of 4 domains to evaluate eligible studies: patient selection, index test, reference standard, flow, and timing. Each domain was assessed in terms of risk of bias, and the first 3 domains were also assessed in terms of concerns regarding applicability. Each item was evaluated as "yes", "no", or "unclear". "Yes" for meeting this criterion, "No" for not meeting this criterion, "unclear" for partially meeting or not getting enough information.

### 2.8. Statistical analysis

The extracted data were analyzed using STATA 11.0 (STATA Corporation, College Station, TX, USA) and Meta-disc 1.4 software. STATA version 11.0 was used for pooling WMD analysis of MPV between groups. Meta-disc software was used to estimate the pooled sensitivity, specificity, positive likelihood ratio (PLR), negative likelihood ratio (NLR), diagnostic odds ratio (DOR), summary receiver operating characteristic (SROC) curve, and the area under the curve (AUC). The *I*^2^ statistics were used to examine statistical heterogeneity between the included studies. The pooled diagnostic value of MPV in the diagnosis of ITP was analyzed using a random-effects model meta-analysis, together with 95% CI. Publication bias was evaluated using Deek’s funnel plot asymmetry test. A *P* value <0.05 was considered statistically significant.

## 3. Result

### 3.1. Study selection

**Fig 1:**
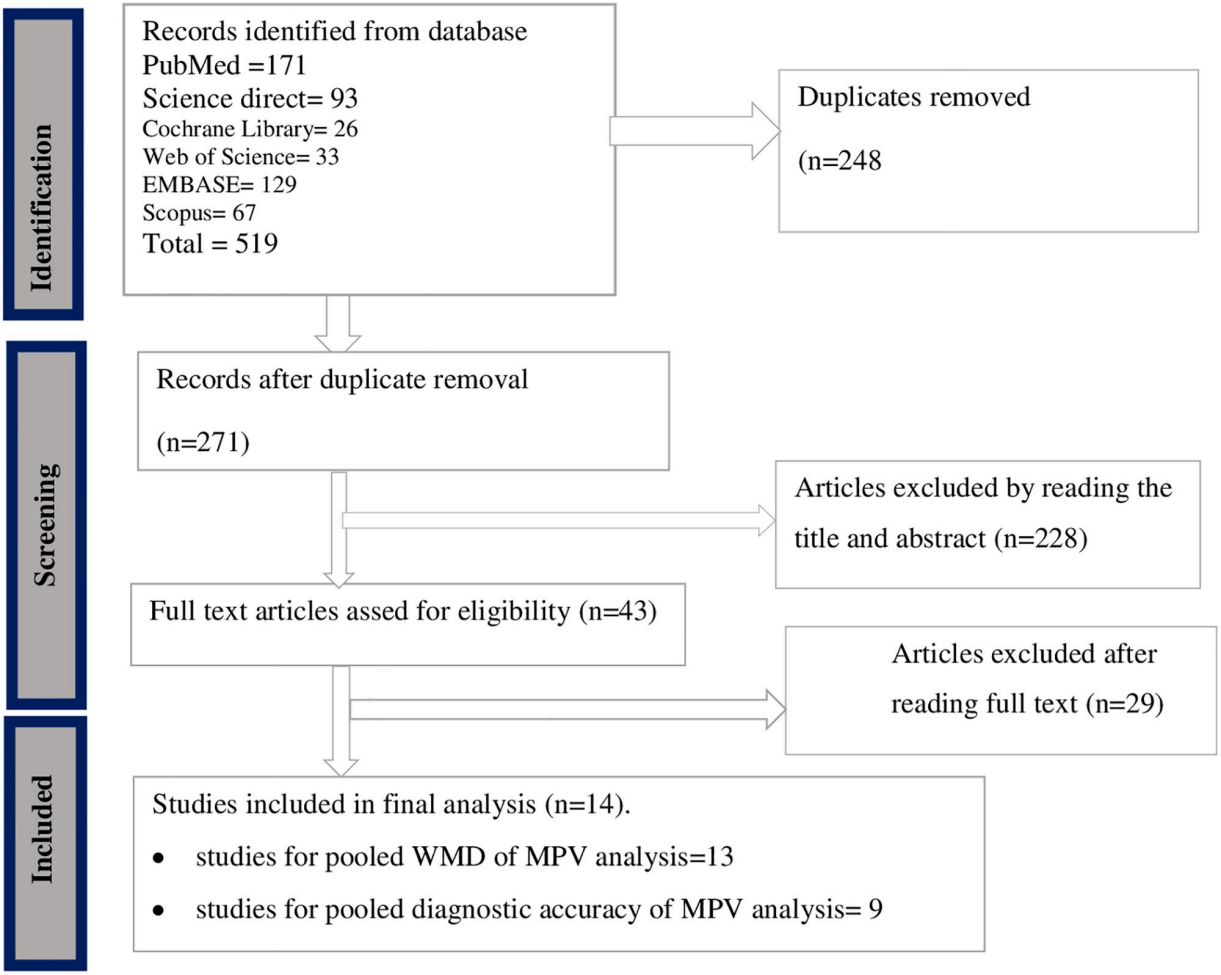
PRISMA 2020 flow chart to describe the selection of studies for the systematic review and meta-analysis on the diagnostic accuracy of MPV in differentiating causes of thrombocytopenia.

A total of 519 studies were identified through database searching which were published up to April 10, 2023, in the English language. Of the identified studies, 248 were removed due to duplicates. Out of the remaining 271 studies, 228 studies were excluded in the title and abstract screening. Moreover, 29 studies were excluded after reading their full text. Finally, 14 articles were included in the meta-analysis after excluding non-relevant articles (Fig 1).

### 3.2. Study characteristics

A total of 14 articles were included in this systematic review and meta-analysis. Out of these, three were conducted in Iraq [34–36], two in Egypt [2, 37], two in China [21, 38], and the other of each were done in Ethiopia [1], Bangladesh [19], Japan [20], Germany [16], Greece [39], Colombia [40], and Pakistan [30] (Table 1).

**Table 1:** Summary characteristics of included studies in the meta-analysis.

Authors	Publication Year	Country	Sample size		MPV value (fl)		Sensitivity (%)	Specificity (%)
			ITP group	hypo-productive group	ITP Group	hypo-productive group		
Niethammer et al [16]	1999	Germany	28	118	10.02± 0.58	8.05± 0.96	96.0	80.0
Kaito et al [20]	2005	Japan	39	40	12.2± 0.2	10.2± 0.2	87.2	80.0
Ntaios et al [39]	2008	Greece	63	71	11.38± 0.57	7.17±0.54	NR	NR
Xu et al [38]	2013	China	124	268	10.3±1.8	9.0±1.8	NR	NR
Aponte-Barrios et al [40]	2014	Colombia	18	36	11.70±1.06	9.82±1.16	94.0	83.0
Elsewefy et al [37]	2014	Egypt	40	40	9.97± 1.35	9.08± 1.25	57.5	82.5
Khaleel et al [34]	2014	Iraq	10	67	12.33± 0.46	10.08±1.81	NR	NR
Negash et al [1]	2016	Ethiopia	33	50	12.4±3.6	9.7±0.9	67.0	95.0
Islam et al [19]	2016	Bangladesh	30	30	12.01±1.23	9.75±1.15	73.3	80.0
Mahdi et al [35]	2017	Iraq	40	50	12.7±2.5	8.2±2.3	90.0	86.0
Tang et al [21]	2017	China	118	35	10.10±1.76	9.4±1.31	74.8	70.3
Al-Sharifi et al [36]	2018	Iraq	39	46	10.39±1.59	9.36±1.19	NR	NR
Mowafy et al [2]	2019	Egypt	15	15	10.1±1.4	9.1±0.9	NR	NR
Khan et al [30]	2020	Pakistan	40	44	NR	NR	59.1	52.9

**Note; ITP**: Immune thrombocytopenia, **MPV**: mean platelet volume, **NR**: not reported

The number of included studies for pooled WMD analysis of MPV between groups was 13 which comprised a total of 597 ITP patients and 866 patients with hypo-productive thrombocytopenia. On the other hand, nine studies that reported sensitivity and specificity were included for pooled diagnostic values of MPV analysis. The total number of participants in this analysis was 829 (386 ITP patients and 443 patients with hypo-productive thrombocytopenia). The diagnostic sensitivity among included studies ranged from 57.5% to 96.0%, while the diagnostic specificity was from 52.9% to 95.0%.

### 3.3. Risk of bias assessment

**Fig 2:**
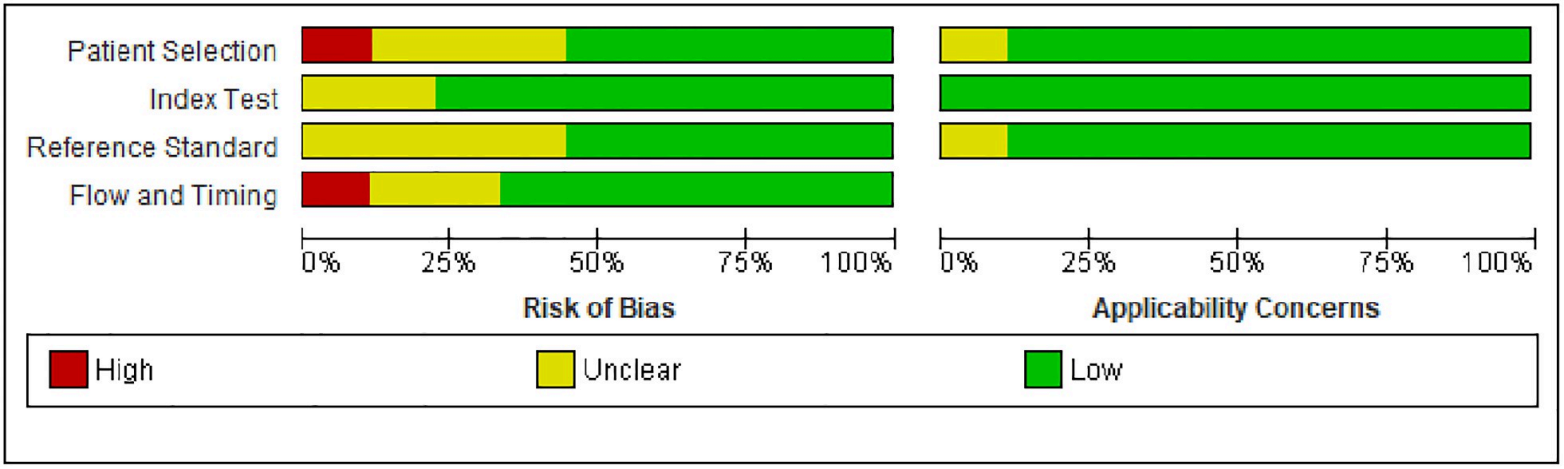
A graph showing risk of bias and applicability concerns among included studies.

The methodological quality assessment for the potential risk of bias and concerns of applicability was done for nine included studies that have been used for the diagnostic value of MPV analysis. The outcomes reveal that the included studies generally satisfied the quality criteria for quality assessment of diagnostic accuracy (Fig 2).

### 3.4. Mean difference of MPV between ITP and hypo-productive thrombocytopenia patients

**Fig 3:**
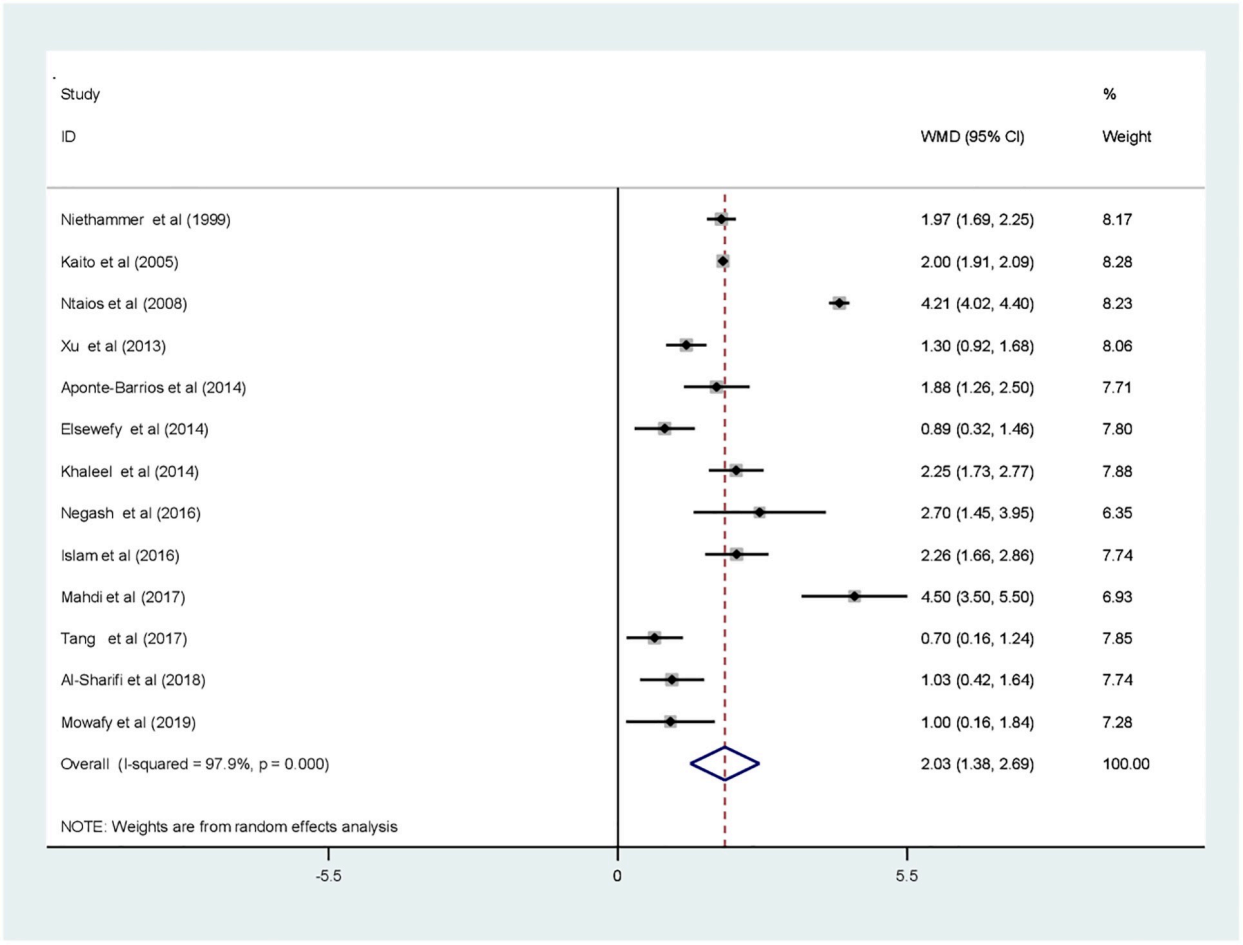
Forest plot of included studies for the pooled WMD estimate of MPV between ITP and HT groups using a random effect model.

The pooled WMD of MPV between the ITP group and the hypo-productive group was analyzed using a random-effects model meta-analysis. This was performed from the included 13 studies that had reported the mean/median value of MPV in both ITP and hypo-productive groups. The overall pooled WMD of MPV between the ITP and hypo-productive groups was 2.03 fl [95% CI; 1.38–2.69], which indicated a significantly increased MPV value in the ITP group as compared to the hypo-productive group. The estimated pooled mean value of MPV in the ITP and hypo-productive groups was 11.17 fl [95% CI; 10.61, 11.74] and 9.14 fl [95% CI; 8.37, 9.93], respectively (Fig 3).

### 3.5. The diagnostic accuracy of MPV

**Fig 4:**
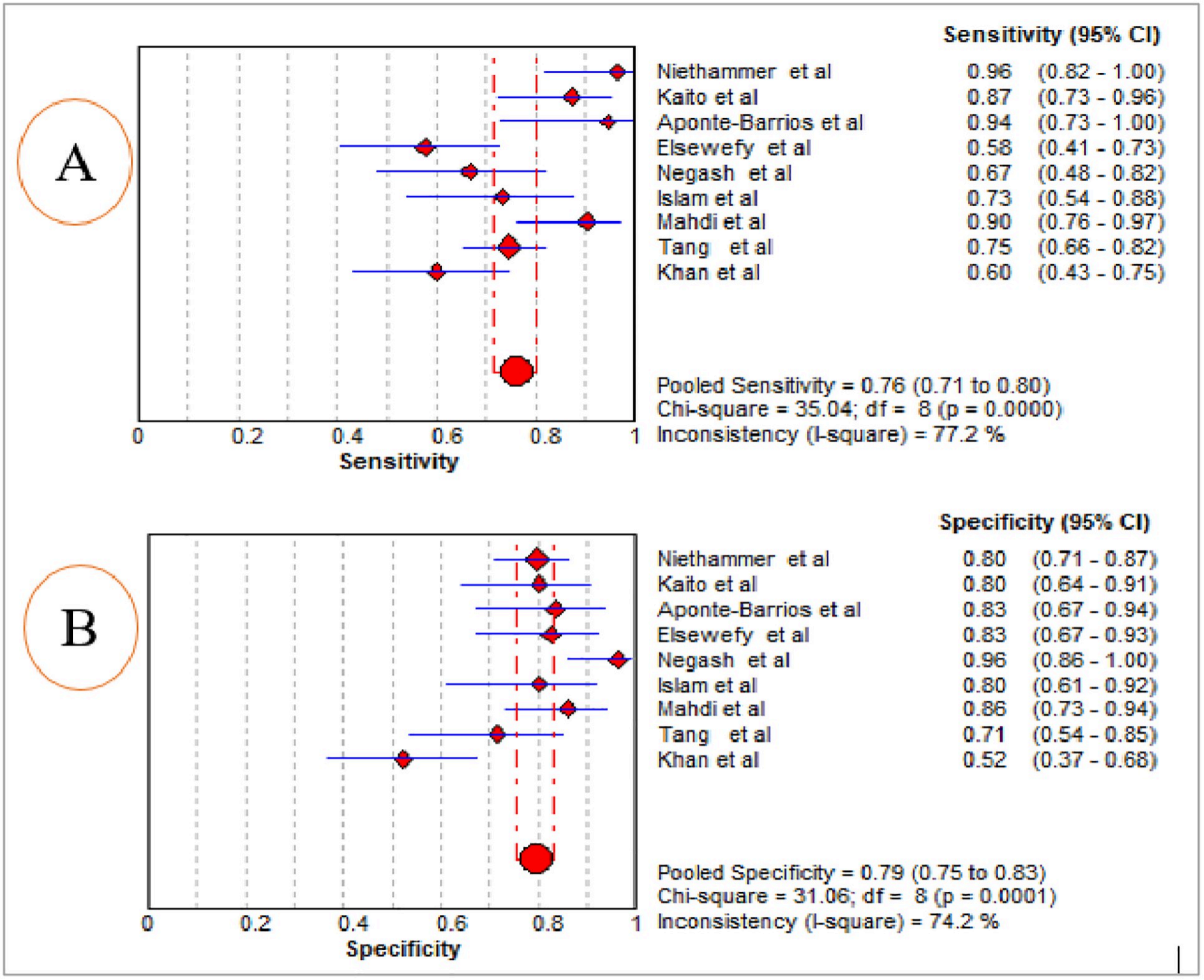
Summarized pooled estimate of sensitivity (A) and specificity (B) of the included studies for the diagnostic accuracy of MPV in the diagnosis of ITP.

**Fig 5:**
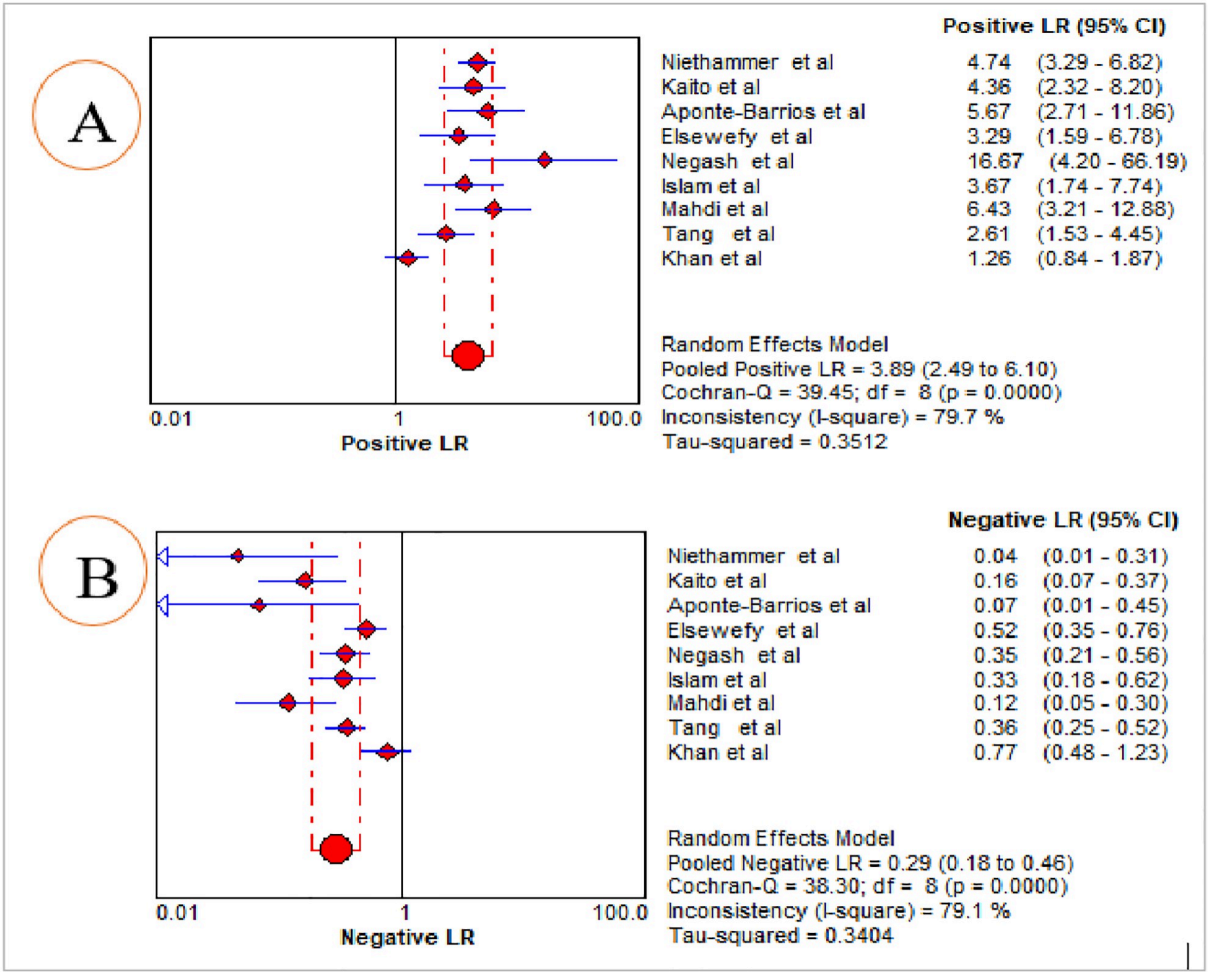
Summary estimate for the pooled PLR (A) and NLR (B) of MPV in the diagnosis of ITP.

**Fig 6:**
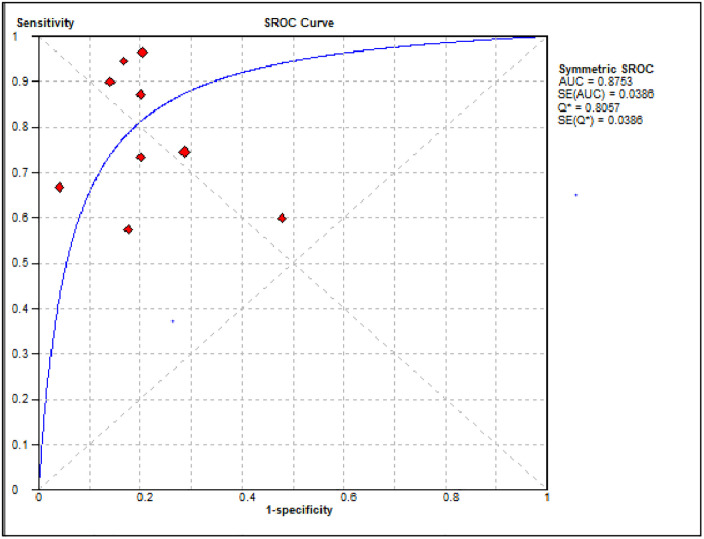
The SROC of the included articles on the systematic review and meta-analysis of the diagnostic accuracy of MPV in the diagnosis of ITP.

**Fig 7:**
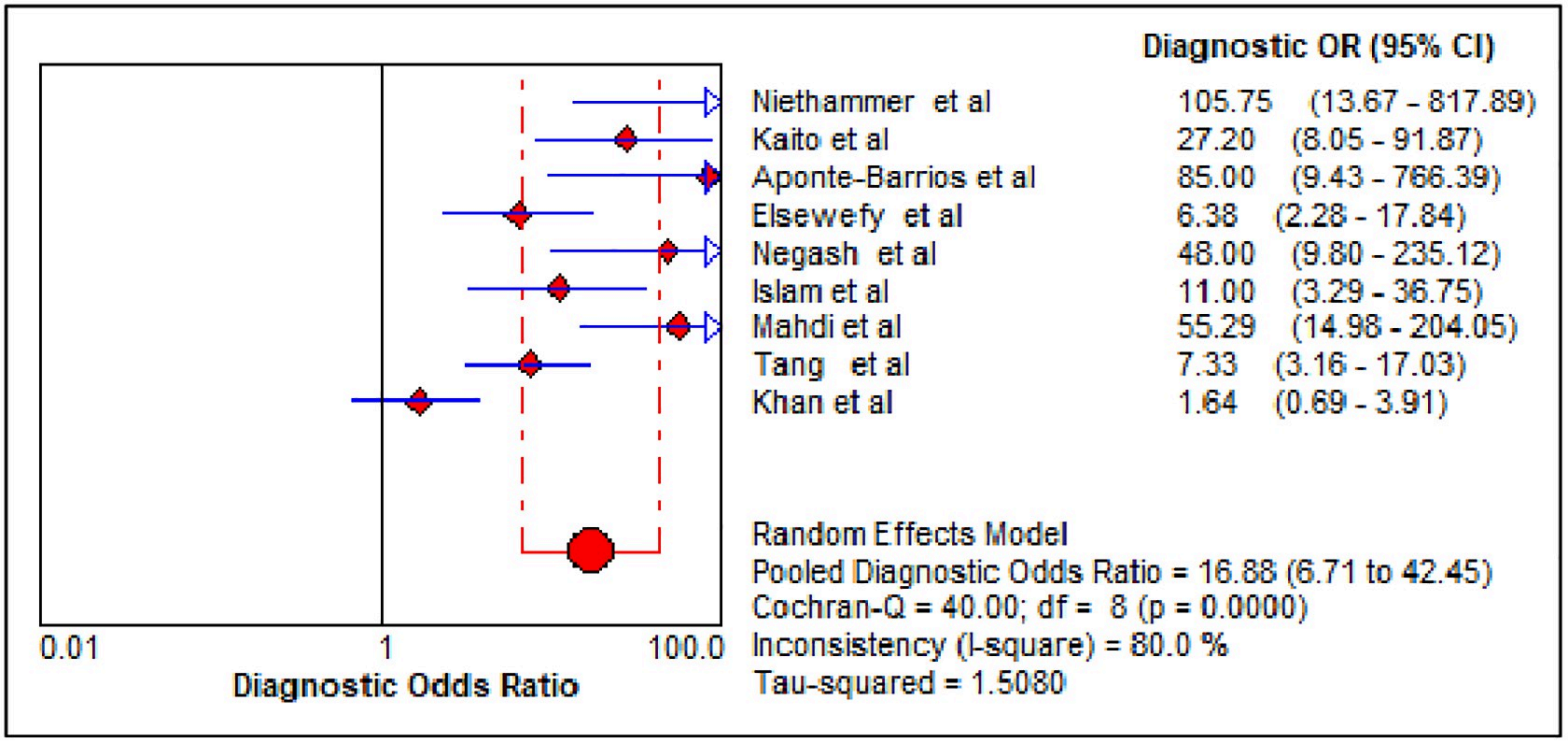
The DOR of the included articles on the systematic review and meta-analysis of the diagnostic accuracy of MPV in the diagnosis of ITP.

The summary diagnostic effectiveness of MPV in differentiating ITP from hypo-productive thrombocytopenia was determined from the included nine studies using the random-effects model. The result showed that the pooled sensitivity and specificity of MPV in differentiating ITP from hypo-productive thrombocytopenia were 76.0% (95% CI: 71.0%, 80.0%) and 79.0% (95% CI: 75.0%, 83.0%), respectively (Fig 4A & 4B). The summary PLR and NLR using the random effects model were 3.89 (95% CI: 2.49, 6.10) and 0.29 (95% CI: 0.18, 0.46), respectively (Fig 5A & 5B). The AUC of the SROC curve was 0.87 and the standard error was 0.0386, suggesting that it was possible to differentiate ITP from hypo-productive thrombocytopenia using MPV with excellent diagnostic performance (Fig 6). Moreover, DOR using the random effects model was 16.88 (95% CI: 6.71, 42.45) (Fig 7).

### 3.6. Publication bias

**Fig 8:**
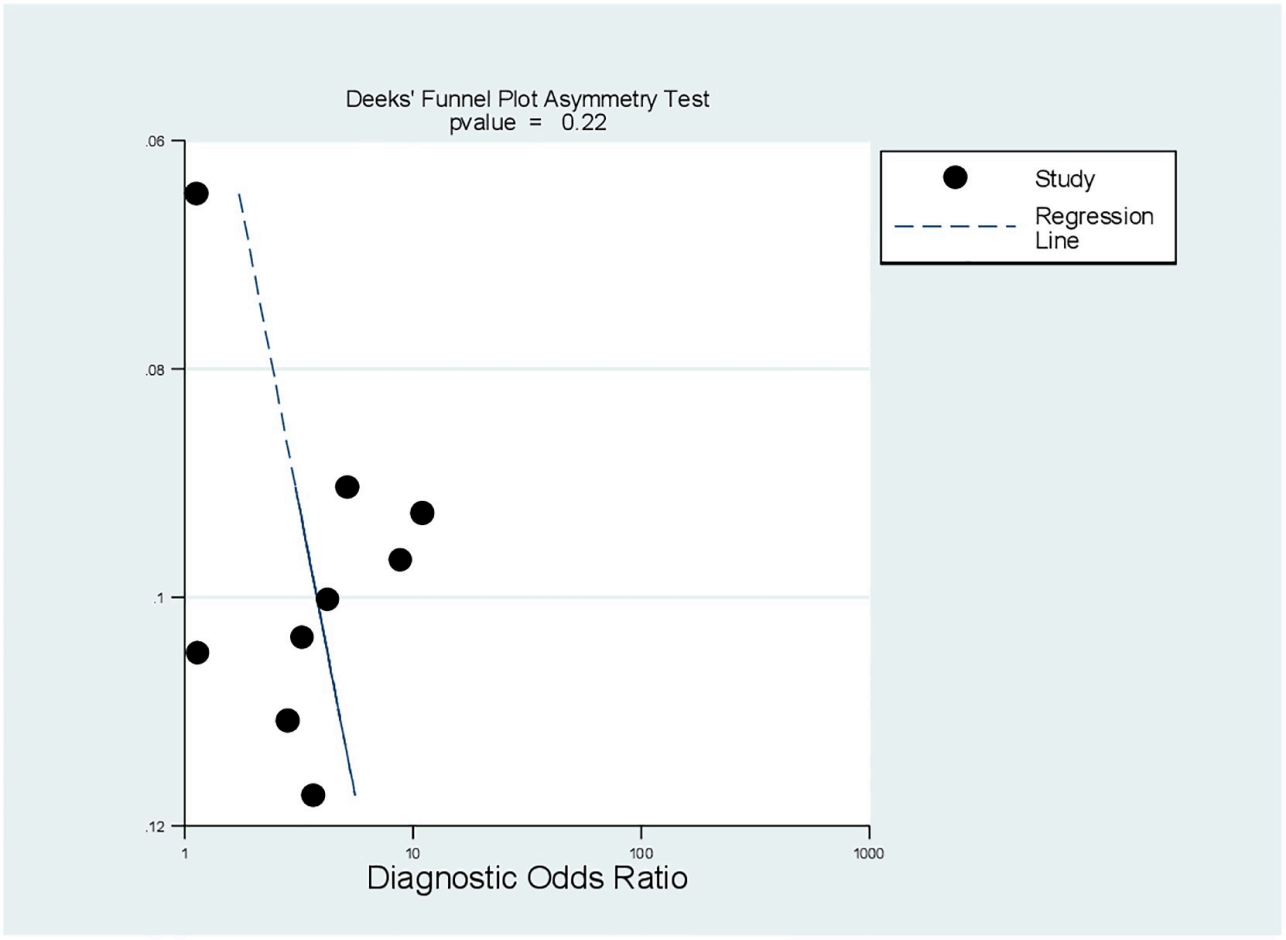
The Deek’s funnel-plot asymmetry test to assess publication bias of included studies.

Deeks’ funnel plot asymmetry test was used to assess the presence/absence of publication bias among nine included studies for the pooled diagnostic value of MPV analysis. Deek’s funnel-plot asymmetry test (a regression of lnDOR against the inverse root of effective sample sizes) has been recommended to assess publication bias in diagnostic accuracy test meta-analyses [41]. *P value* <0.10 for the test of the correlation coefficient indicating significant asymmetry. However, the P value in this review indicated that there was no proof of potential publication bias among the included studies (P = 0.22) (Fig 8).

## 4. Discussion

Thrombocytopenia is a common clinical manifestation of several disorders and potentially one of the most life-threatening diseases [42]. It has numerous causes which are often divided into two major causes: decreased bone marrow production which results in hypo-productive thrombocytopenia and accelerated destruction of platelets which results in hyper-destructive thrombocytopenia [37]. Whereas, hyper-destructive thrombocytopenia mainly occurs as a result of an immune-mediated process known as ITP [6]. ITP is an autoimmune bleeding disorder that primarily manifests as megakaryocyte maturation disorder in the bone marrow and increased platelet destruction in the peripheral blood. The management of ITP is guided by an adequate knowledge of the cause and accurate diagnosis [8].

Laboratory indicators help for better diagnosis of ITP and adequate drug treatment [8]. However, the diagnosis of ITP is still challenging due to the lack of clear biomarkers that can tell for sure if a person has ITP [8]. The major concern in diagnosing ITP is distinguishing ITP from other causes of thrombocytopenia, which often have a similar presentation but may require completely different management approaches [43]. Therefore, it is important to have a diagnostic tool that helps to distinguish accurately whether thrombocytopenia in a patient is a result of ITP or hypo-production of platelets [20, 35, 40]. The availability of automated hematology analyzers with recent advances in technology has made it possible to record various platelet indices which is quick and easily available in a clinic [40]. There is a growing interest in the use of platelet markers in discriminating different causes of thrombocytopenia [1, 39]. As a result, the platelet-derived indices of MPV which provide important information regarding platelet kinetics have been suggested as a good diagnostic marker in discriminating ITP from hypo-productive thrombocytopenia [15].

In this study, the pooled WMD of MPV between the ITP group and hypo-productive group was performed using a random-effects model meta-analysis from the included 13 studies. The result indicated a significantly increased MPV value in the ITP patients as compared to HP patients (WMD = 2.03; 95% CI, 1.38–2.69). The result suggests that MPV value has been increased in patients with ITP which may be a result of a relative increase in young platelets [29]. An increased new platelet production in the bone marrow leads to the release of larger reticulated platelets into the bloodstream, resulting in a high MPV in ITP [22]. On the contrary, patients with HT have decreased values of MPV due to old and small platelets [39, 44].

A total of nine studies were included in this systematic review and meta-analysis to determine the pooled diagnostic accuracy of MPV in differentiating ITP from hypo-productive thrombocytopenia. The result revealed that the pooled sensitivity and specificity of MPV in differentiating ITP from hypo-productive thrombocytopenia were 76.0% (95% CI: 71.0%, 80.0%) and 79.0% (95% CI: 75.0%, 83.0%), respectively. The AUC of the SROC curve was 0.87, suggesting that it was possible to differentiate ITP from hypo-productive thrombocytopenia using MPV with excellent diagnostic performance. The result has been reflected due to an increased value of MPV secondary to peripheral destruction, reflecting the release of young platelets [34, 45]. Thus, MPV could help to distinguish ITP from hypo-productive thrombocytopenia [20], which may avoid unnecessary invasive bone marrow aspiration [1].

## 5. Strengths and limitations of the study

The strength of this review is that a comprehensive search was done on different databases using different search strategies. The study included all relevant articles done around the globe. Furthermore, the review was done following the protocol of the PRISMA guideline and critical appraisal of the methodological quality of the included studies was done using the QUADAS-2 tool which is recommended for diagnostic tests quality assessment. However, some limitations remain in this study. The high degree of heterogeneity in the included studies may limit the interpretation of the study. Moreover, most of the included studies are from the Middle East, which may influence the representativeness of the pooled estimate.

## 6. Conclusion

Data from the studies analyzed in this review showed that MPV is significantly higher in ITP patients compared to patients with hypo-productive thrombocytopenia. Moreover, the pooled diagnostic accuracy estimate in this review suggests the utility of MPV in diagnosing ITP d patients. Therefore, the MPV derived from the CBC test could be an easily measurable marker for frequent diagnosis and treatment monitor, and it would easily be scaled up in resource-limited settings due to its widespread availability.

## Supporting information

S1 Checklist PRISMA check list 2020. (DOCX)

## Abbreviations

AUC: Area under the curve
CBC: Complete blood count
DOR: Diagnostic odds ratio
GP: Glycoprotein
IQR: Interquartile range
ITP: Immune thrombocytopenia
MDS: Myelodysplastic syndrome
MPV: Mean platelet volume
NLR: Negative likelihood ratio
PAIgG: Platelet-associated immunoglobulin G
PLR: Positive likelihood ratio
PRISMA: Preferred reporting item for systematic review and meta-analysis
PROSPERO: Prospective register of systematic reviews
QUADAS-2: Quality assessment of diagnostic accuracy studies 2
SD: Standard deviation
SROC: Summary receiver operating characteristic
WMD: Weighted mean difference
